# Sex differences in the efficacy and tolerability of antipsychotic drugs in people with an acute exacerbation of schizophrenia: protocol for an individual-participant-data, dose-response, network meta-analysis

**DOI:** 10.12688/f1000research.169873.1

**Published:** 2025-09-23

**Authors:** Spyridon Siafis, Yuki Furukawa, Johannes Schneider-Thoma, Nobuyuki Nomura, Natalia E. Fares-Otero, Bram W. C. Storosum, Jasper B. Zantvoord, Christian Schwarz, Julia Schulz, Horst Naiser, Karl Heinz Möhrmann, Josef Priller, John M. Davis, Orestis Efthimiou, Georgia Salanti, Stefan Leucht

**Affiliations:** 1 Technical University of Munich, TUM School of Medicine and Health, Department of Psychiatry and Psychotherapy, Munich, Germany; 2 German Center for Mental Health (DZPG), partner site Munich-Augsburg, Germany; 3 Department of Neuropsychiatry, University of Tokyo, Tokyo, Japan; 4 Department of Neuropsychiatry, Keio University School of Medicine, Tokyo, Japan; 5 Department of Psychiatry and Psychology, Bipolar and Depressive Disorders Unit, Hospital Clínic, Institute of Neurosciences, University of Barcelona (UBNeuro), Fundació Clínic per a la Recerca Biomèdica, Institut d'Investigacions Biomèdiques August Pi i Sunyer (FCRB-IDIBAPS), Barcelona, Spain; 6 Department of Psychiatry, Amsterdam UMC, Amsterdam Neuroscience, Location Academic Medical Centre, University of Amsterdam, Amsterdam, The Netherlands; 7 BASTA-Bündnis für psychisch erkrankte Menschen, Munich, Germany; 8 Landesverband Bayern der Angehörigen psychisch erkrankter Menschen e.V., Munich, Germany; 9 Neuropsychiatry, Charité-Universitätsmedizin Berlin and Deutsches Zentrum für Neurodegenerative Störungen, 10117, Berlin, Germany; 10 University of Edinburgh and UK DRI, Edinburgh, UK; 11 Psychiatric Institute, University of Illinois at Chicago, Chicago, USA; 12 Johns Hopkins University, Baltimore, USA; 13 Institute of Primary Health Care (BIHAM), University of Bern, Bern, Switzerland; 14 Institute of Social and Preventive Medicine (ISPM), University of Bern, Bern, Switzerland

**Keywords:** Antipsychotics; psychosis; sex differences; systematic review; IPD meta-analysis; precision psychiatry

## Abstract

**Background:**

There are currently no sex-specific recommendations for antipsychotic prescribing in schizophrenia, although women may be more susceptible to certain side-effects and may require lower doses to achieve comparable efficacy to men. However, sex differences remain insufficiently studied to inform treatment decisions.

**Methods:**

We plan a systematic review and individual-participant-data meta-analysis of randomized trials comparing different antipsychotic drugs, doses, formulations with each other or with placebo in adults with acute schizophrenia. Eligible studies will be identified through the Vivli platform, and their individual-participant-data will be harmonized into a common dataset. Two independent reviewers will screen search results and assess risk of bias using the Risk of Bias 2 tool. The primary outcome will be overall symptoms of schizophrenia, and secondary outcomes will include a broad range of efficacy, tolerability, acceptability, and dosing measures.

A stepwise approach to data synthesis will be adopted, depending on individual-participant-data availability, aiming to examine sex differences in the effects of antipsychotic drugs compared to placebo. If sufficient data are available, we will conduct random-effects meta-analyses using regression models to evaluate biological sex, typically categorised as male or female, as both a prognostic factor and an effect modifier, network meta-analyses to synthesize direct and indirect evidence, and dose-response meta-analyses using restricted cubic splines to explore dose-effect relationships. The robustness of the findings will be assessed through sensitivity analyses adjusting for additional covariates.

The certainty of evidence on sex differences will be evaluated using the Grading of Recommendations Assessment, Development and Evaluation (GRADE) approach for subgroup analysis. If the certainty is judged to be moderate or high, sex-specific treatment effects will be further assessed using the Confidence in Network Meta-Analysis (CINeMA) framework.

**Discussion:**

This study will examine sex differences in the efficacy, tolerability, and dosing of antipsychotics in acute schizophrenia, potentially contributing to evidence-based, sex-specific treatment recommendations for antipsychotic prescribing.

**Protocol registration**

PROSPERO-ID: CRD420251022957

## Introduction

Antipsychotic drugs are the mainstay of treatment in schizophrenia, ^1^ and sex can influence their risk-to-benefit ratio. ^2– 4^ Women and men differ in body composition, physiology, and lifestyle, which in turn affect the pharmacokinetics and pharmacodynamics of these medications. ^2– 5^ As a result, women may be more susceptible to certain side-effects (e.g., weight gain, prolactin elevation) and may require lower doses to achieve comparable efficacy, potentially due to smaller average body size and weight, resulting in higher plasma concentrations at equivalent doses. ^2– 5^ Yet sex differences remain poorly characterized, and current treatment guidelines lack evidence-based, sex-specific recommendations. ^2– 4, 6, 7^

There is also a lack of comprehensive synthesis on this topic. Individual studies are typically underpowered to detect subgroup differences and yield exploratory and preliminary findings. ^8– 11^ Systematic reviews and meta-analyses based on group-level data, even those designed to assess subgroup responses, ^12, 13^ are inconclusive due to limited sex-stratified reporting. Although complex and resource-intensive, individual-participant-data (IPD) meta-analyses can overcome these challenges. Two previous IPD meta-analyses suggested that women, on average, respond better to antipsychotics than men ^14, 15^; however, they did not assess key clinical questions related to specific drugs, dosing, or side-effects. This gap is relevant to clinical practice, as dose-response meta-analyses showed that while efficacy plateaus at around 4-6 mg/day risperidone equivalents, many side-effects continue to increase with higher doses. ^16– 20^

### Objectives

To address this evidence gap, we will conduct a systematic review and synthesis of IPD from randomized trials in the Vivli ^21^ database that compared antipsychotics with each other or placebo in individuals with acute schizophrenia. The aim is to examine sex differences in efficacy, tolerability and dosing using robust meta-analytic methods and input from a multidisciplinary team including clinicians, statisticians, and people with lived experience. The findings may inform sex-specific recommendations in treatment guidelines.

We will focus on subgroup differences based on biological sex, typically categorized as female or male, which is a key variable influencing drug effects. ^22^ Gender, as a sociocultural construct, may also affect treatment outcomes; however, it is beyond the primary aims of this project, and most included trials were likely conducted before distinctions between sex and gender were widely recognized or systematically captured in clinical research.

## Protocol

This research project involves an independent secondary analysis of an existing anonymized dataset from already completed clinical trials, accessed through the Vivli platform. ^21^ The project was registered on 22.04.2025 with PROSPERO (ID: CRD420251022957) and the protocol is reported according to the PRISMA statement for protocols (PRISMA-P) (see extended data). ^23^

We follow the Sex and Gender Equity in Research (SAGER) guidelines (see extended data). ^22^ As the project focuses on subgroup differences based on biological sex (see “Objectives”), we will use the terms “female” and “male” individuals in the technical parts of the protocol (see also Table 1 for the definitions of sex and gender), but also refer to “women” and “men” to support broader contextualization in relation to clinical practice and existing literature.

**Table 1.** Table of tentative list of variables.

	Variable	Level	Comment
**Study identification and characteristics**	Study name	Study	The study name (e.g., last author and publication year or a numerical identifier) will be used for study identification.
	Publication year	Study	The year the study was published. If it is not published, the year of study completion or the year the results were first made publicly available will be recorded.
	Sponsor	Study	The name of the industry or sponsor that conducted or funded the study.
	Number of sites	Study	The number of sites in the trial.
	Study site identifier	Individual	An identifier for each of the sites in a trial.
	Country	Individual	The country in which each study site is located.
	Trial design	Study	Parallel or crossover trial (only the first phase will be considered for crossover trials).
	Blinding	Study and outcome	Double-blind (participants and outcome assessors are blinded to the treatment), single-blind (outcome assessors are blinded to the treatment), open-label (neither participants nor outcome assessors are blinded). Single-blinded and open trials will be excluded in a sensitivity analysis.
	Study duration	Study	The duration of the treatment in a trial. Trials should have a minimum duration of three weeks.
	Risk of bias	Study and outcome	We will use RoB2 ^31^ to assign domain-specific and overall judgments of the risk of bias for each study. Studies with a high risk of bias in the randomization process will be excluded from the review. Eligible studies with an overall high risk of bias will be excluded in a sensitivity analysis.
**Arm identification and intervention characteristics**	Arm identifier	Arm	An identifier for each of the arms in a trial.
	Drug name	Arm	The name of the drug to which individuals were assigned, including antipsychotics and placebo, will be recorded. We will use the generic names of the drugs as presented in the Anatomical Therapeutic Chemical classification.
	Drug group	Arm	In addition to analyses of any antipsychotic and individual antipsychotics, we will consider grouping drugs with similar mechanisms of action to increase statistical power. We will use a data-driven taxonomy based on receptor-binding profiles, which groups antipsychotics into four categories ^32^: drugs with muscarinic M2–M5 receptor antagonism, dopamine D2 partial antagonism and adrenergic antagonism, serotonergic and dopaminergic antagonism, and dopaminergic antagonism.
	Route of administration	Arm	Oral or long-acting injectable formulations. Different formulations of the same drug will be considered as the same intervention in the main analysis, and as distinct interventions in a sensitivity analysis.
	Dosing schedule	Arm	Fixed or flexible dosing schedule.
	Dose	Arm and individual	Dose information at both the arm and individual level will be considered. When possible, we will use the daily dose (mg/day) as randomized, including fixed doses or the median of the dose range in flexible-dose studies. Alternative estimations will be applied, when necessary, in flexible-dose designs. When pooling different antipsychotics or formulations, doses will be converted into a common metric using established dose equivalents. ^19^
**Participant identification and characteristics**	Participant identifier	Individual	An identifier for each of the participants in a trial.
	Age	Individual	Age in years at the start of the trial will be considered. We will explore potential nonlinear relationships to account for menopausal status in women. ^2, 11^ Although we will seek this information, we do not expect menopausal status to be commonly reported in the trials, and estimation based on age may be necessary.
	Sex and gender	Individual	We will focus on biological, typically categorized as female and male, as it is a key biological variable that can influence drug effects. ^22^ We will prefer the definition of sex assigned at birth; however, variable definitions across the included studies are expected. These will be accepted, and the definition used in each study will be recorded. Many of the included studies were likely conducted before clear distinctions between sex (as a biological construct) and gender (as a sociocultural construct) were widely recognized or systematically documented in clinical research. As a result, trial participants were likely often classified based on self-report, rather than through confirmed biological determination (e.g., karyotyping) or even a standardized record of sex assigned at birth. Moreover, data on gender, understood as a sociocultural construct existing along a spectrum, are not expected to have been widely collected in the included studies. If such data are available, we will record and report them accordingly.
	Race/ethnicity	Individual	Race or ethnicity will be recorded as reported in each study, although such data are not expected to be consistently available. When reported, these variables will be used to better characterize the study populations. If included in any analyses examining sex differences, we will acknowledge that race and ethnicity are sociocultural constructs that are related to other socioeconomic factors.
	Body size	Individual	Body size can affect the pharmacokinetics of the drugs, and women have on average a smaller body size. ^2, 5^ We will consider height (m), weight (kg) and body mass index (kg/m ^2^). In the analysis, we will prefer body mass index, estimated from height and weight if not reported, but if not, enough data are available, we will use weight.
	Diagnosis	Individual	We will consider the diagnostic terms of the schizophrenia spectrum used to include participants in the trial. Particular attention will be given to the classification of diagnoses as schizophrenia or schizoaffective disorder, as the latter is more frequent in women and often requires concomitant treatments (e.g., antiepileptics), which may influence the effects of antipsychotics. ^5, 7^
	Diagnostic criteria	Individual	We will consider the methods of diagnosis, whether operationalized with specific diagnostic criteria such as DSM-5 or ICD-11, or based on clinical judgment. Studies without operationalized criteria will be excluded in the sensitivity analysis.
	Age of onset and duration of illness	Individual	Age of onset and duration of illness can influence the outcomes, and women with schizophrenia generally have a later onset than men. ^5, 7, 15^ Age of onset and duration of illness are closely related and correlated with each other and with age. These interrelationships will be considered when including this variable in the analysis.
	Smoking status and other substance use	Individual	Smoking might influence the effects of the drugs, and men with schizophrenia are more frequently smokers than women. ^5^ The inclusion of this variable, as well as any specification of the coding and necessary transformations, will be considered a posteriori, depending on the availability and consistency of data across studies (e.g., whether smoking is reported as a binary variable such as current smoker vs. non-smoker or with more detailed measures such as cumulative exposure or frequency). Although misuse or abuse of substances other than smoking may be an important variable influencing outcomes and potentially exhibiting sex differences, individuals with substance use are often excluded from trials. Therefore, we do not expect to be able to include information on this variable.
**Outcomes**	See “Outcome” section	Individual	We will consider data on a comprehensive set of efficacy, tolerability, and acceptability outcomes, assessed at baseline and across multiple time points during the trial.

### Eligibility criteria

Studies meeting the following eligibility criteria in terms of study design, participants and interventions will be included in the systematic review.

### Study design

We will include blinded and open randomized controlled-trials (RCTs) comparing eligible antipsychotic drugs, doses, or formulations (see “Interventions”) with each other or with placebo as monotherapy for acute schizophrenia. Trials must have a minimum treatment duration of three weeks. ^24^

The first phase of crossover trials will be considered to avoid carryover effects. ^25^ We will exclude studies with high risk of bias in the randomization process (see “Study risk of bias”), and cluster-randomized trials due to unit-of-analysis concerns. ^25^

There will be no other restrictions in terms of publication year, publication status, sponsorship, language or country.

*Population*

Adults with acute schizophrenia, schizophreniform disorder, or schizoaffective disorder will be eligible. There will be no upper age limit or restrictions based on sex, setting, ethnicity, or diagnostic criteria. Studies in which fewer than 20% of participants have a different mental health condition will be included, and all participants with schizophrenia-spectrum disorders will be eligible for the IPD analysis.

We will exclude studies focusing specifically on the following subgroups: children or adolescents, advanced age, comorbid somatic illness or substance use disorders, first-episode psychosis, treatment resistance, predominant negative symptoms, or clinical stability or remission at baseline. This approach is consistent with previous meta-analyses ^1^ to safeguard the transitivity assumption. However, if enough studies in patient subgroups are identified, we will request access to these for potential secondary analyses and separate publications.

### Interventions

We will include monotherapy with second-generation antipsychotics (i.e., amisulpride, aripiprazole, asenapine, brexpiprazole, cariprazine, clozapine, iloperidone, lumateperone, lurasidone, olanzapine, quetiapine, paliperidone, risperidone, sertindole, ziprasidone, and zotepine), three first-generation antipsychotics (i.e., chlorpromazine, haloperidol, and perphenazine), and placebo. Other first-generation antipsychotics will be excluded, as they have been studied in smaller, lower-quality trials and are no longer in use in most industrialized countries. ^1, 26^

There will be no dose restrictions, as the aim is to examine whether women require lower doses than men and because optimal dosing in women remains unclear. ^3, 27^ We will include both fixed- and flexible-dose trials, in which investigators titrate to the appropriate dose. For flexible-dose trials, we will use the median of the randomized dose range in the main analysis and conduct sensitivity analyses using alternative estimates and by excluding these studies.

Oral and long-acting injectable antipsychotics will be considered the same intervention in the main analysis and as separate interventions in a sensitivity analysis. We will exclude short-acting injectables used for the management of acute agitation, as well as studies involving augmentation or combination strategies with antipsychotics.

### Study identification and selection

*Study identification*

We will search for all studies available in Vivli, ^21^ a major data-sharing platform that includes published and unpublished trials conducted by both pharmaceutical companies and academic institutions. Studies from YODA, ^28^ another data-sharing platform primarily containing Johnson & Johnson trials, are also accessible through Vivli. The search will be conducted through the NIAID Data Ecosystem by downloading all available studies in Vivli, and data requests will be submitted via Vivli and YODA as appropriate.

We will not search for trials that are not already available in Vivli, as the hundreds of potentially eligible RCTs, ^1^ would require resources beyond the scope of this project. However, if feasible, we will consider complementing our analyses with data requested from the Dutch regulatory authority, as these were used in a previous similar analysis. ^14^

*Study selection*

Two independent reviewers will screen all titles and abstracts of available records in Vivli and retrieve full texts for studies considered relevant or unclear. This process will be facilitated by our previous meta-analyses of group-level data from acute schizophrenia trials. ^1^ Discrepancies will be resolved through discussion or, if needed, by a third senior reviewer. If uncertainty persists, we will contact Vivli for clarification. Study selection will be presented in a PRISMA flowchart. ^29, 30^

### Data collection process

*Data harmonization and integrity*

IPD from eligible RCTs will be requested and obtained through Vivli, harmonized, and merged into a common dataset accompanied by a data dictionary. We will consider data on study identification, methodology, population and interventions relevant for study description and analysis (see Table 1 for a tentative list of variables), as well as outcome data across different time points (see “Outcomes”) and risk of bias assessments (see “Study risk of bias”).

Data integrity will be assessed by checking for missing values, duplicates, outliers, and verifying the appropriateness of randomization. To ensure internal consistency, IPD from each study will be summarized into aggregate form and cross-checked against summary statistics from published reports, which are expected to be available and were double-extracted in our previous meta-analysis. ^1^ Any discrepancies will be addressed through discussion or by contacting Vivli.

*Study risk of bias*

Two independent reviewers will evaluate risk of bias using the Risk of Bias 2 (RoB2) ^31^ tool, following the approach used in our previous meta-analyses. ^1, 33^ RoB2 evaluates potential biases in the randomization process, deviations from intended interventions, missing outcome data, outcome measurement, and selection of reported results, and provides an overall risk-of-bias judgment. ^31^ The domain of missing outcome data will be assessed based on the IPD after imputation, rather than relying on published reports.

### Outcomes

We will consider a comprehensive set of efficacy, acceptability, and tolerability outcomes relevant for investigating sex differences, selected based on previous meta-analyses of antipsychotics ^1^ and refined with input from clinicians and individuals with lived experience.

*Primary outcome*

The primary outcome will be overall symptom of schizophrenia measured with the Positive and Negative Syndrome Scale (PANSS) ^34^ or Brief Psychiatric Rating Scale (BPRS) ^35^ or any other validated scale. ^36^ To have the same outcome measure across trials in the IPD analysis, BPRS data will be converted to PANSS by a validated method of equipercentile linking. ^37^ If other scales are used, data will be synthesized using standardized mean differences (see “Effect sizes”), a method that will also be applied to other outcomes assessed with different instruments.

*Secondary outcomes*

We will examine the following secondary outcomes:

- Response to treatment defined by ≥50% reduction of PANSS/BPRS, which corresponds to at least “much improvement” according to the global impression of clinicians, and is recommended in acutely-ill patients. ^38^
- Positive, negative and depressive symptoms of schizophrenia measured with validated rating scales (e.g., PANSS sub-scores).
- Quality of life and functioning measured with validated rating scales.
- Clinical Global Impression including severity (CGI-S) and improvement (CGI-I) scales. ^39^
- Premature discontinuation (dropout) due to any reason, inefficacy and side-effects.
- Mortality due to any reason.
- Side-effects commonly associated with antipsychotics ^1^ including akathisia, extrapyramidal symptoms (e.g., parkinsonism, dystonia, and dyskinesia, or use of antiparkinsonian medications as a proxy), anticholinergic side-effects (e.g., blurred vision and dry mouth), sedation, QTc interval prolongation, weight increase and other metabolic disturbances (e.g., glucose and lipid abnormalities), prolactin elevation (ng/ml) and associated side-effects (e.g., sexual side-effects and disturbances in menstruation). Given the variability in how adverse events are measured in clinical trials, we will aim to harmonize the available data using the Medical Dictionary for Regulatory Activities (MedDRA). ^40^ Where appropriate, continuous measures will be transformed into dichotomous outcomes using established thresholds (e.g., ≥7% weight gain from baseline or validated cut-offs from adverse event or movement disorder scales), though preference will be given to continuous measures when available.
- Blood levels of antipsychotics (ng/L) measured with validated methods. ^41^

*Effect sizes*

For continuous outcomes, we will use mean differences (e.g., total PANSS scores, weight in kg) or standardized mean differences (SMD) when multiple scales are used to assess the same construct.

For dichotomous outcomes, we will use odds ratios (OR) due to their favourable mathematical properties in meta-analysis, ^42^ and convert them into absolute and relative risks for better interpretability, using the pooled absolute risk in the placebo group as the control event rate. ^43^

For time-to-event outcomes, we will use hazard ratios if estimable from the available data, otherwise, we will treat them as dichotomous.

*Timing*

The time point of measurement will be as close to 6 weeks as possible, which is the typical duration in acute schizophrenia trials, ^1^ with a minimum follow-up of 3 weeks (see “Study design”). Adverse events may occur at any time during the follow-up period.

We will use data from baseline and all available time points in the statistical modelling or for multiple imputation, and we will consider longer-term outcomes if available.

### Data synthesis

*Synthesis approach*

The main aim of the analysis is to examine differences between women and men across outcomes when comparing antipsychotic to placebo, analysed according to randomized allocation. As noted in the introduction, our *a priori* hypothesis is women may be more susceptible to side-effects and may require lower doses to achieve comparable efficacy.

We will adopt a multi-step strategy, progressing from simpler to more fine-grained synthesis methods. We will begin by presenting sex-disaggregated summary data for each study, followed by pairwise meta-analyses comparing antipsychotics with placebo, dose-response meta-analyses, and if feasible, dose-response network meta-analyses. ^44^

Sex differences will be explored initially for antipsychotics as a group, and if data permit, at the level of individual drugs and/or classes according to their receptor-binding profiles. ^32^ Similarly, we will first assess sex differences irrespective of antipsychotic dose. If sufficient data are available, we will conduct dose-specific analyses by categorizing doses into groups (e.g., informed by previous dose-response meta-analysis ^19^ or expert consensus) ^27^ or, preferably, by modelling dose-response relationships using restricted cubic splines, with knot placement informed by quantiles of the available dose distributions. ^45^ If needed when pooling different antipsychotics, their doses will be converted to a common scale using dose equivalents. ^19^

Evidence for sex differences is expected to be drawn primarily from comparisons within trials that include both sexes; however, data from trials that enrolled only one sex will also be considered. We will first conduct stratified analyses for females and males. If sufficient data are available, we will perform regression models that include sex both as a prognostic factor (i.e., associated with outcomes irrespective of treatment) and as an effect modifier (i.e., influencing the relative treatment effect). Adjustment for additional prognostic factors will be considered in sensitivity analyses, particularly for age (accounting for potential nonlinear relationships related to menopausal status), baseline severity, and body size (e.g., weight and height, which may influence plasma drug levels), as well as other sociodemographic or clinical characteristics, depending on availability across studies (see Table 1). ^2– 4^

The final selection of the synthesis approach, including the choice between one- or two-stage IPD meta-analysis, Bayesian or frequentist frameworks, regression model specifications, and variable standardization, will be determined *a posteriori*, based on data availability across studies, their clinical relevance, and input from clinicians, statisticians, and individuals with lived experience.

*Heterogeneity, transitivity assumption and incoherence*

We will conduct random-effects meta-analyses to account for between-study heterogeneity, with heterogeneity quantified using the between-study variance (τ-squared), assumed to be common across treatment comparisons in network meta-analysis, ^25^ and 95% prediction intervals. In case of rare events, we will consider appropriate methods for their analysis. ^46^

In network meta-analysis, the transitivity assumption is essential for valid indirect comparisons. ^25, 47^ To support this, we will apply strict eligibility criteria to ensure that included studies are sufficiently similar and that interventions could, in principle, be jointly randomized. We will further assess transitivity by examining the distribution of potential effect modifiers (e.g., age, sex, country, age of onset, duration of illness, baseline severity) across treatment comparisons. Incoherence, i.e., statistical disagreement between direct and indirect evidence, will be evaluated using both local (i.e., separate indirect and direct evidence) and global approaches (i.e., design-by-treatment interaction test). ^47^

*Missing outcome and covariate data*

Missing outcome and covariate data will be addressed using multiple imputation, accounting for trial-specific stratification of patients and assuming data are missing at random unless there is evidence suggests otherwise. ^48^ Multiple imputed datasets will be generated and analysed separately, with results pooled using Rubin’s rules. ^49^

For multiple measurements, mixed models of repeated measurements will be considered.

*Sensitivity analyses*

We will conduct sensitivity analyses to assess the robustness of the findings for the primary outcome by: a) adjusting for additional prognostic factors that may influence sex differences; b) excluding flexible-dosing studies to rule out potential bias introduced by clinicians prescribing lower doses to women or using alternative estimates of the dosing; c) excluding studies that did not use operationalized diagnostic criteria; d) excluding open-label and single-blind studies; e) excluding studies with a high overall risk of bias; and f ) treating oral and long-acting injectable formulations of the same antipsychotic as distinct interventions.

*Reporting and data availability bias*

Sex differences in antipsychotic effects are not expected to be a source of publication or data availability bias. However, we will assess small-study effects using funnel plots, ^50^ and by including study variance as a covariate in the model. Potential data availability bias will be explored by comparing the characteristics of studies available in Vivli with those that are not, using aggregate data already available previous meta-analyses. ^1^

*Confidence in the evidence*

We will assess the confidence in the evidence for sex differences in the effects of antipsychotics for the following outcomes: overall symptoms, weight gain, prolactin levels, QTc interval prolongation, extrapyramidal side-effects, akathisia, sedation, and anticholinergic side-effects.

The credibility of subgroup effects will be assessed using the Grading of Recommendations Assessment, Development and Evaluation (GRADE) approach for subgroup analyses ^51^ and the Instrument to assess the Credibility of Effect Modification Analyses (ICEMAN). ^52^ This approach applies an algorithm based on signalling questions related to the design and analysis of the subgroup comparison, the consistency of effect modification across trials, and other relevant factors. Based on these considerations, the credibility of subgroup effects will be rated as very low, low, moderate, or high, corresponding to judgments ranging from “very likely no effect modification” to “very likely effect modification”. ^51, 52^

If the credibility of sex differences is judged as moderate or high, we will present sex-specific treatment effects and evaluate the confidence in the evidence using the Confidence in Network Meta-Analysis (CINeMA) framework, ^53^ considering six domains of bias: within-study bias (assessed using RoB2), ^31^ indirectness (expected to be low given the strict eligibility criteria of the included studies), reporting bias (see “Reporting and data availability bias“), heterogeneity, imprecision and incoherence (evaluated by comparing the 95% confidence/credible intervals and 95% prediction intervals against predefined equivalence ranges of |SMDs|<0.1 and ORs 0.83-1.20).

If the credibility of sex differences is judged as very low or low, we will present the overall effects alongside the sex-specific treatment effects for completeness and indicate that treatment decisions should primarily be guided by the overall population estimates, as established by previous comprehensive meta-analyses of aggregate data. ^1^

*Statistical software*

The analysis will be conducted in R statistical software ^54^ using appropriate packages such as meta, ^55^ netmeta, ^56^ dosresmeta ^57^ and crossnma, ^58^ and self-programmed routines in JAGS ^59, 60^ within the Vivli secure computing environment. ^21^

### Patient and public involvement

People with lived experience of schizophrenia and/or current antipsychotic treatment, both women and men, their relatives, and representatives from the patient and family organizations *Bündnis für psychisch erkrankte Menschen* (BASTA) and *Aktionsgemeinschaft der Angehörigen psychisch Kranker* (ApK e.V. Bavaria) are actively involved in all stages of the project, including reactor, advisory, and leadership roles. Patient and public involvement will be reported in accordance with the GRIPP2 short-form checklist. ^61^

During the conceptualization phase and preparation of the funding application (2023–2024), we held joint discussions with individuals with lived experience who identified the investigation of sex differences in antipsychotic effects as a high-priority topic. They contributed to key elements of the study design, including the definition of inclusion criteria and selection of outcomes, with particular emphasis on examining dose-effects and adverse events in addition to efficacy.

To ensure a multidisciplinary perspective, we established a steering committee composed of clinicians, methodologists, statisticians, and individuals with lived experience (listed as co-authors or acknowledged in the protocol). The committee held its kick-off meeting at the start of the project, where the study protocol was discussed, including the selection of outcomes and covariates potentially influencing sex differences (see Table 1), as well as the rationale for a stepwise synthesis strategy (see “Data synthesis”). The committee meets biannually to support progress across all stages of the review, including the interpretation of findings and the joint dissemination of results in accessible language together with individuals with lived experience.

### Dissemination of information

We plan to publish the systematic review and meta-analysis as open access in peer-reviewed journals, potentially resulting in multiple publications, and to present the findings at scientific conferences. The results will be reported in accordance with the PRISMA statement ^29^ and its extensions for network meta-analyses ^62^ and IPD, ^30^ as well as the SAGER guidelines. ^22^ Lay summaries will be prepared and disseminated with the support of patient and caregiver groups (see “Patient and Public Involvement”). The findings are expected to inform clinical treatment guidelines and digital tools for shared-decision making. ^63, 64^ To support future research, we will make the aggregated data generated during the analysis freely available, including sex-disaggregated summary data for all outcomes in each study. This will be done in accordance with the FAIR principles, ^65^ and we will comply with any terms outlined in the data use agreements with Vivli and YODA.

### Study status

As of the date of the first submission of this protocol on 27.08.2025, we have completed the formal study identification in Vivli and initiated, but not yet completed, the screening of search results against the inclusion criteria, the receipt of IPD, and data harmonization. We have not yet commenced data synthesis.

As noted in the PROSPERO registration, a preliminary search in Vivli identified 44 trials (18,568 participants) on eight antipsychotics (aripiprazole, haloperidol, lurasidone, olanzapine, paliperidone, quetiapine, risperidone, and ziprasidone) and placebo. Following project initiation and ongoing screening, no new eligible studies have been found, and IPD from a few trials are no longer available, as anonymized data including information on sex are not accessible. The current estimate includes 37 studies (15,750 participants) for the above-mentioned interventions except for lurasidone, with additional data potentially identified after screening is complete.

There were no changes from the PROSPERO registration, except for further specification and clarification of the methods, such as a clearer presentation of the data synthesis approach and the assessment of confidence in the evidence.

There were some changes from earlier versions of the protocol registered on Vivli and YODA, which were prepared during the funding application process. Specifically, we will not apply any restrictions in the eligibility criteria based on the dose of antipsychotics, as examining dose effects was deemed a crucial component of the data synthesis. In addition, we revised the tentative list of variables ( Table 1) to focus on core factors, whereas earlier versions included an extended list containing variables that may not be considered in the current analysis due to lower relevance and/or limited expected availability across studies (e.g., current living situation, employment status).

Any deviations or additional specifications will be reported and explained in the manuscript and reflected in updates to the PROSPERO registration.

## Discussion

This systematic review and meta-analysis aims to provide the first robust evidence on sex differences in the effects of antipsychotic doses versus placebo across efficacy, acceptability, and tolerability outcomes, with the goal of informing long-awaited sex-specific treatment recommendations. ^2, 6^ This will be achieved by synthesizing IPD from a large and representative sample of 37 studies (15,750 participants), possibly the largest dataset in schizophrenia research to date. The study will be supported by a multidisciplinary team of clinicians, researchers, statisticians, and individuals with lived experience to ensure rigorous data analysis and selection of clinically relevant outcomes and covariates.

Treatment guidelines for antipsychotic prescribing are largely based on clinical trials in which men are overrepresented, and their uniform applicability to both women and men has been increasingly questioned. ^2, 3, 5, 66^ Previous research has suggested sex differences in the effects of antipsychotics, but most findings are based on narrative reviews, ^2, 4, 5^ systematic reviews with qualitative synthesis, ^3, 13^ secondary analyses of individual studies. ^8– 11^ Two previous IPD meta-analyses also showed that SMDs for antipsychotics versus placebo were, on average, 0.05-0.10 larger in women than in men. ^14, 15^ However, these studies did not examine sex differences in side-effects or at the drug- or dose-specific level, which are also highly relevant, given indications that women may be more susceptible to certain side-effects (e.g., weight gain and prolactin elevation) and may require lower doses for comparable efficacy (e.g., due to smaller body size). ^2– 5, 8– 10, 13^ Our analysis will build on this literature by applying advanced meta-analytic methods (e.g., network meta-analysis, dose-response meta-analysis, and regression) to provide a quantitative synthesis estimating the magnitude and certainty of sex differences across a broad range of outcomes related to efficacy, tolerability, acceptability, and dosing.

We anticipate certain limitations and challenges. Only studies available in the Vivli database will be included, but we expect limited reporting biases (see “Reporting and data availability bias”) and a representative set of dopamine D _2_ receptor-blocking antipsychotics covering a range of mechanisms of action and side-effect profiles: haloperidol, risperidone, and paliperidone (dopamine and serotonin antagonism, associated with movement disorders and prolactin elevation); olanzapine and quetiapine (muscarinic M _2-5_ receptor antagonism, associated with anticholinergic side-effects and weight gain); and ziprasidone and aripiprazole (dopamine D _2_ receptor partial agonism and/or adrenergic antagonism, with a relatively benign side-effect profile). However, as with any IPD meta-analysis, it will not be possible to include all available evidence (>400 trials, >30 compounds), ^1^ and data for individual antipsychotics may be insufficient to examine drug-specific effects.

Although a high degree of standardization is expected in the IPD across trials in Vivli, potential variability in data availability and reporting limits the ability to define the data synthesis approach *a priori.* We outlined a tentative variable list ( Table 1) and a stepwise synthesis strategy; however, final decisions on coding, transformations, and synthesis will be made *a posteriori*, based on the harmonized data and prior to commencing data analysis.

Finally, we will focus on differences based on biological sex, but we anticipate variability in its definition across the included studies, many of which were conducted before clear distinctions between sex (as a biological construct) and gender (as a sociocultural construct) were widely recognized or systematically documented in clinical research (see Table 1). We also acknowledge that gender identities, as sociocultural constructs, may also influence treatment preferences, experiences, and outcomes (e.g., adherence, lifestyle) ^67^; however, these cannot be examined with the available data. In addition, we will not be able to investigate sex differences in the pharmacokinetics (e.g., absorption, distribution, metabolism, and excretion) or pharmacodynamics (e.g., receptor occupancy) of antipsychotics, ^2, 67^ as such data are often not reported in clinical trials.

In conclusion, our planned systematic review and IPD meta-analysis will be the first to comprehensively examine sex differences in the efficacy, tolerability, and dosing of antipsychotics in acute schizophrenia. By synthesizing IPD from a large sample using advanced meta-analytic methods, this study has the potential to provide evidence-based insights to inform sex-specific treatment recommendations for antipsychotic prescribing.

## Article information

### Ethics and consent

The project received approval from the Technical University of Munich ethics committee (2025-270-S-NP). This research project involves an independent secondary analysis of an existing anonymized dataset from already completed clinical trials, accessed through the Vivli platform. Vivli’s policy is to only accept anonymized data. It is the responsibility of the data contributors to ensure that they can share the data being requested and meet all global regulations and policy before sharing the data. The data contributors have conducted all necessary checks during the data contributor review.

## Data Availability

No data associated with this article. Zenodo: Protocol for meta-analysis on sex differences in antipsychotic effects (APSEDI). https://doi.org/10.5281/zenodo.16942378 . ^68^ This project contains the following extended data:
- PRISMA-P checklist.pdf - SAGER checklist.pdf Data are available under the terms of the Creative Commons Attribution 4.0 International license (CC-BY 4.0). See Reporting Guidelines in the appendix that will be added in a repository. Zenodo: PRISMA-P checklist for “Protocol for meta-analysis on sex differences in antipsychotic effects (APSEDI)”. https://doi.org/10.5281/zenodo.16942378 . ^68^ Zenodo: SAGER checklist for “Protocol for meta-analysis on sex differences in antipsychotic effects (APSEDI)”. https://doi.org/10.5281/zenodo.16942378 . ^68^ Data are available under the terms of the Creative Commons Attribution 4.0 International license (CC-BY 4.0).
