# Supplementary material for: Sex differences in the efficacy and tolerability of antipsychotic drugs in people with an acute exacerbation of schizophrenia: protocol for an individual-participant-data, dose-response, network meta-analysis
Source: F1000Res. 2025 Sep 23;14:967. [Version 1] doi: 10.12688/f1000research.169873.1 (PMC12824477; doi:10.12688/f1000research.169873.1)
Supplement: Peer review history

# Reviewer report: Reviewer response for version 1

**DOI:** 10.5256/f1000research.187258.r436552

**Published:** 2026-01-21

**Authors:** Rosa Catalán

**Affiliations:** 1 Barcelona Clinic Schizophrenia Unit (BCSU), Neurosciences Institute, Hospital Clínic of Barcelona, University of Barcelona, IDIBAPS, Barcelona, Spain

This protocol addresses a clinically important question with clear implications for practice guidelines: potential sex differences in antipsychotic efficacy, tolerability, and dosing in acute schizophrenia. The methodological plan is strong and ambitious—IPD, NMA, and dose–response meta-analysis using restricted cubic splines—alongside credibility assessment (GRADE, ICEMAN) and confidence in network meta-analysis (CINeMA). Adherence to PRISMA-P and SAGER and meaningful patient and public involvement (PPI) are notable strengths.

 My recommendation is to approve it with a slight minor revision. Below I outline strengths and areas for improvement.

 Strengths

 Its clinical relevance and novelty given the lack of sex-specific prescribing recommendations and inconclusive prior evidence fully justify this study. Moreover, the scope and the design are appropriate to my opinion. Focusing on acute exacerbation to safeguard transitivity and including common SGAs and FGAs, placebo, and oral/LAI formulations, the authors would be able to provide relevant and reliable results. They are aligned with PRISMA-P and SAGER; meaningful PPI described.

 Areas needing improvement

 - Maybe authors may consider adding a supplemental Statistical Analysis Plan (SAP) detailing:

 - Exact models (formulae) for the primary outcome and sex×treatment interactions.

 - A priori decisions on spline knot placement (e.g., 10–50–90th percentiles) and number of knots based on data availability.

 - Criteria to choose between two-stage vs one-stage, and frequentist vs Bayesian, with a decision flowchart.

 - Inclusion of scale minima is excellent but BPRS details are missing. It would be useful that authors explicitly state the BPRS minimum used and cite the equipercentile linking reference; include sensitivity with alternative response definitions (e.g., ≥20% in less severe patients).

Is the study design appropriate for the research question?

Yes

Is the rationale for, and objectives of, the study clearly described?

Yes

Are sufficient details of the methods provided to allow replication by others?

Yes

Are the datasets clearly presented in a useable and accessible format?

Yes

Reviewer Expertise:

Psychiatry, schizophrenia, psychopharmacology , pharmacogenetics of antipsychotics

I confirm that I have read this submission and believe that I have an appropriate level of expertise to confirm that it is of an acceptable scientific standard.

# Reviewer report: Reviewer response for version 1

**DOI:** 10.5256/f1000research.187258.r443741

**Published:** 2025-12-27

**Authors:** Mete Ercis

**Affiliations:** 1 Mayo Clinic, Rochester, MN, USA

This manuscript is a protocol for a systematic review and individual-participant-data meta-analysis of randomized trials assessing sex differences in the efficacy, tolerability, and dosing of antipsychotic medications in adults with schizophrenia and schizoaffective disorders. The study aims to generate robust evidence to inform potential sex-specific treatment recommendations.

 It is a very well-written, methodologically rigorous, and timely. The rationale is clear, the objectives are well defined, and to my knowledge, the planned methods reflect best practices. My only comment is that the finalized manuscript could benefit from briefly expanding the description of the Vivli platform, as some readers may not be familiar with it.

Is the study design appropriate for the research question?

Yes

Is the rationale for, and objectives of, the study clearly described?

Yes

Are sufficient details of the methods provided to allow replication by others?

Yes

Are the datasets clearly presented in a useable and accessible format?

Yes

Reviewer Expertise:

Psychiatry; psychopharmacology; schizophrenia and mood disorders; clinical epidemiology.

I confirm that I have read this submission and believe that I have an appropriate level of expertise to confirm that it is of an acceptable scientific standard.
